# Paroxysmal Sympathetic Hyperactivity After Traumatic Brain Injury: What Is Important to Know?

**DOI:** 10.7759/cureus.24693

**Published:** 2022-05-03

**Authors:** Sidi Mamoun Louraoui, Fadwa Fliyou, Jehanne Aasfara, Abdessamad El Azhari

**Affiliations:** 1 Neurosurgical Department, Mohammed VI Hospital, Casablanca, MAR; 2 Department of Neurology, Cheikh Khalifa International University Hospital, Faculty of Medicine, Mohammed VI University of Health Sciences (UM6SS), Casablanca, MAR

**Keywords:** psh, dysautonomia, sympathetic storm, traumatic brain injury, paroxysmal sympathetic hyperactivity

## Abstract

Traumatic brain injury (TBI) is one of the leading causes of morbidity and mortality. The etiologies of TBI are varied and its complications can lead to paroxysmal sympathetic hyperactivity that was first described as a “sympathetic storm” or “diencephalic autonomic seizure.” The clinical manifestations are rapid and sudden onset of sympathetic hyperactivity characterized by tachycardia, systolic hypertension, hyperthermia, tachypnea, and diaphoresis, all summarized in the latest and most accepted diagnostic criteria. The pathophysiology remains controversial with many theories proposed. Given the clinical manifestations, the complications, outcomes, and lack of popularity of the syndrome, we report a case treated in our institution and review the current pathophysiology and treatment options.

## Introduction

Traumatic brain injury (TBI) is one of the leading causes of morbidity and mortality worldwide, specifically in developing countries where its incidence remains high (7.9 million cases/year) [1]. The etiologies of TBI are varied, and its pathophysiology can lead to a large array of clinical symptoms such as paroxysmal sympathetic hyperactivity (PSH), seen in 80% of the cases of TBI [1,2].

Wilder Penfield first described the clinical features of PSH after TBI in 1929 considering that it likely had an epileptic cause [1,3]. He named the syndrome mesencephalic seizure or diencephalic autonomic seizure [4]. Since then, in a 2010 review of 349 cases, 31 different terms for the syndrome were found in the published literature. The terms were defined either based on the description of clinical symptoms, the assumed epileptic pattern, the location, or the neurological damage [3,4].

The lack of clear definition and adequate terminology explains the underrecognition of the syndrome despite its high incidence, as well as the slow progress in understanding its pathophysiology [1,3]. For many years, physicians failed to distinguish the association between parasympathetic and sympathetic hyperactivity and pure sympathetic hyperactivity [4]. In 2010, the term PSH was approved as the unifying terminology of this condition. In addition, the definition and the diagnostic criteria were established, as follows: a complication of various acute brain lesions irrespective of the cause (traumatic or other) that result in a disturbance of the central regulation of autonomic function, thus excluding clinical syndromes with a parasympathetic component [3,5,6].

Given the lack of popularity of the syndrome and the impact that PSH might have on the outcome of patients, we report a case of PSH after a TBI and review the pathophysiology, clinical symptoms, management, and prognosis of this frequent but unknown pathology [2,6].

## Case presentation

**Figure 1:**
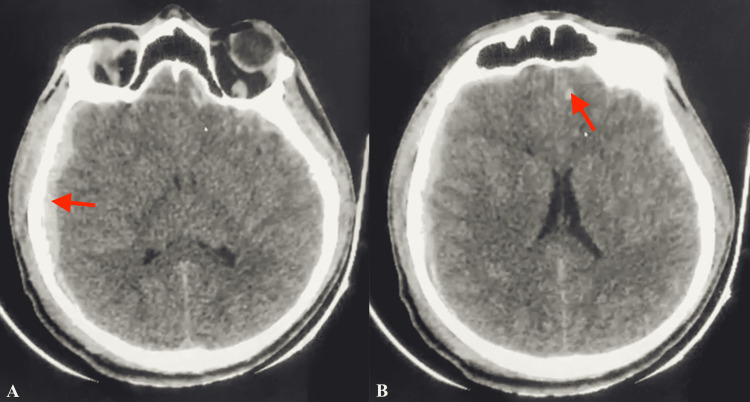
Preoperative axial non-injected CT scan showing the subdural hematoma, brain edema, and frontal contusions. A: Red arrow showing the fronto-temporal-occipital subdural hematoma. B: Red arrow showing the frontal contusion and brain edema. CT: computed tomography

A 21-year-old male was admitted to the emergency department in the aftermath of a traffic car accident. On physical examination two hours after the incident, the patient had a Glasgow Coma Scale (GCS) score of 7/15 (E1V1M5), right anisocoria, and ipsilateral ear bleeding. The patient was hemodynamically stable, and the rest of the somatic examination was unremarkable. A body computed tomography (CT) scan revealed a right 10 mm temporoparietal subdural hematoma, diffuse brain swelling, multiple left temporoparietal contusions, and a right petrous bone fracture. No other lesions were noted on the CT scan (Figure 1).

The patient benefitted from a right fronto-temporal-parietal-occipital decompressive craniectomy allowing hematoma evacuation and coagulation of the bleeding veins responsible for the hematoma with dural enlargement for brain swelling treatment. The bone flap was addressed for cryo-conservation. He was transferred to the intensive care unit for further care. The postoperative course was satisfactory, and the patient started showing signs of recovery. However, on postoperative day three, the patient presented with repeated episodes of transitory bilateral mydriasis, tachycardia, hypertension, tachypnea, fever, exaggerated diaphoresis, and dystonic posturing.

A head CT scan showed an increase in the temporal contusions and brain swelling; however, no hemorrhagic transformation was noted. Electroencephalography showed no signs of epileptic activity.

While remaining unresponsive most of the time, the patient’s external stimulation (whether noxious or non-noxious) triggered the same chain of events with the episodes being sudden, rapid, and resolving spontaneously within a few minutes.

The diagnosis of PSH was validated according to the PSH assessment score combining the clinical features scale and diagnosis likelihood tool with a score of over 17 [3-5]. The patient benefited from medical treatment using 300 mg of gabapentin per day at first, followed by 600 mg per day with complete resolution of the episodes after four days. His neurological status improved over the weeks allowing him to regain full consciousness six weeks after the injury. He started motor re-education to improve his autonomy. He then benefited from the second surgery for his bone flap replacement. Recovery was rapid after surgery and the patient was discharged a few days later. Follow-up at three months showed no complications and no signs of PSH. The degression of gabapentin was planned progressively.

## Discussion

In 2010, Perkes et al. published the first review of 349 PSH case reports and reported that 80% followed TBI, 10% followed anoxic brain injuries, 5% followed stokes, and 5% followed hydrocephalous, hypoglycemia, infections, or unspecified causes [4]. The prevalence of PSH after TBI ranges from 8% to 33% [5-7].

For many decades, the pathophysiology of PSH remained poorly understood. Even until a few years ago, the mechanism was not completely understood. It has now clearly been separated from autonomic dysreflexia (uncontrolled elevation in systolic blood pressure subsequent to injuries in the spinal cord at or above the sixth thoracic vertebrae) and parasympathetic hyperactivity [8].

**Figure 2:**
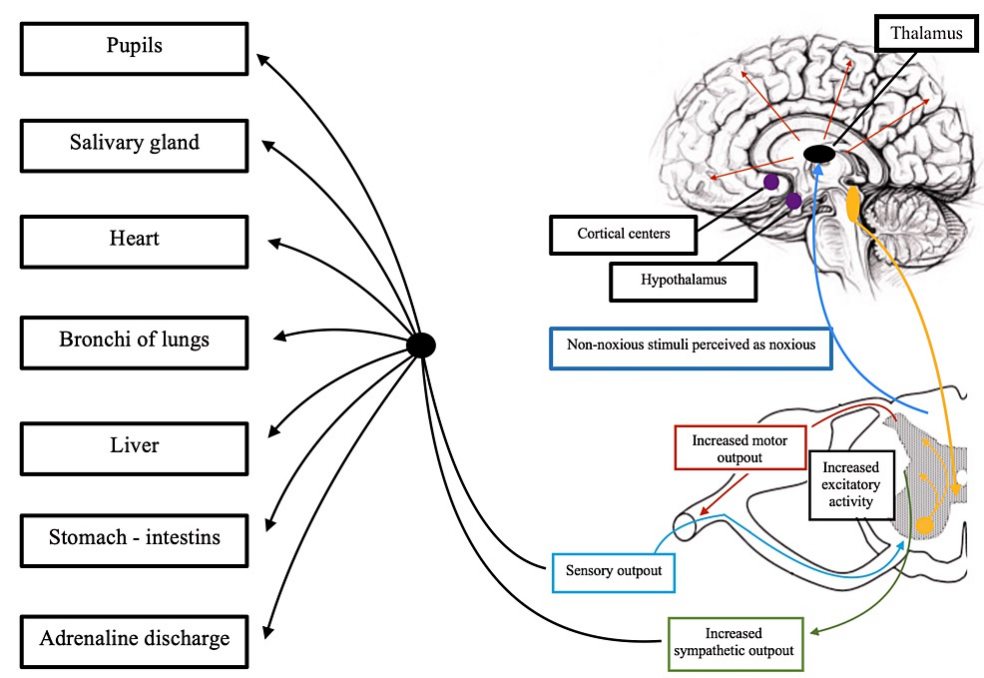
PSH pathophysiology showing the summary of the physiology of the sympathetic hyperactivity according to the excitatory/inhibitory ratio model and the impact on the organs. PSH: paroxysmal sympathetic hyperactivity

Several mechanisms have been considered to explain the loss of inhibition of the sympathetic nervous system without parasympathetic involvement [1,3,8]. The first is the epileptogenic theory which considered epileptic discharges to be the cause of sympathetic inhibition loss. However, because most anti-epileptic treatments are ineffective, the theory was rapidly abandoned [1,3]. Second, the disconnection theory hypothesized that there is a sympathetic stimulation in the brainstem, hypothalamus, and spinal cord without inhibition from the cortical structures (hippocampus, amygdala, insular cortex, cingulate cortex, middle temporal cortex, and dorsolateral prefrontal cortex) [1]. The damages sustained during TBI to the inhibitory centers lead to a disconnection of these centers from caudal excitatory centers; however, it does not explain the paroxysmal pathway [1]. The third theory speculated that neuroendocrine regulation disturbances might be responsible for PSH; uncontrolled adrenergic outflow with massive unload (200-300%) of catecholamines that arise from increased excitability of the central sympathetic nervous system [1,3,8]. The last and most accepted theory according to the literature is the excitatory/inhibitory ratio model which considers PSH to be a two-stage process. First, a sympathetic excitation from the subcortical structure and a lack of inhibitory pathways (disconnection or destruction). After a delay, the patient recovers the inhibitory factors which explain the sympathetic trigger by external stimuli [1,3,8] (Figure 2).

Although several studies have tried to determine the exact location of the lesions that are responsible for PSH, they have failed to show that patients who developed PSH after TBI might be associated with diffuse axonal injury, focal parenchymal lesions, midbrain or pontine lesions, and paraventricular white matter or corpus callosum lesions [3].

As described in the case described here, the clinical manifestations are complex. It comprises cardinal symptoms that can either be all present at the onset, or the patient could present with one symptom or a combination of symptoms. This wide clinical manifestation is explained by the individual differences and the use of some drugs as a treatment for TBI that can mask some symptoms. It also explains the lack of diagnosis in some cases or the delay in diagnosis. Clinical manifestations are characterized by a transient simultaneous rapid and sudden increase in sympathetic and motor activity triggered by external stimulation: tachycardia, hypertension, tachypnea, diaphoresis, dystonic posturing, and hyperthermia. The following additional symptoms have also been described: pupillary dilatation, reduced level of consciousness, flushing, horripilation, and agitation [1,6]. Between 1993 and 2008, many diagnostic criteria were published. In 2014, an international consensus produced the diagnostic criteria used today; it selected 11 of the 16 items to be pathognomonic of PSH. It separated the first criteria to determine the severity of the PSH syndrome and the second to determine the likelihood of the presence of PSH (Table 1) [3-5].

**Table 1:** Paroxysmal sympathetic hyperactivity assessment measure.

Clinical feature scale (CSF)					
	0	1	2	3	Score
Heart rate (beats per minute)	<100	100–119	120–139	>140	
Respiratory rate (breaths per minute)	<18	18–23	24–29	>30	
Systolic blood pressure (mmHg)	<140	140–159	160–179	>180	
Temperature (°C)	<37	37–37.9	38–38.9	>39	
Sweating	Nil	Mild	Moderate	Severe	
Posturing during episode	Nil	Mild	Moderate	Severe	
			CSF subtotal		
Diagnosis likelihood tool (DLT) – scored as 1 if present					
Clinical features occur simultaneously					
Episodes are paroxysmal in nature					
Sympathetic over-reactivity to normally non-painful stimuli					
Features persist for >3 consecutive days					
Features persist for >2 weeks post-brain injury					
Features persist despite treatment of alternative diagnosis					
Medication administered to decrease sympathetic features					
>2 episodes					
Absence of parasympathetic features during episodes					
Absence of other presumed cause of features					
Antecedent acquired brain injury					
			DLT subtotal		
Combined total (CSF + DLT)					
		Unlikely			<8
PSH diagnostic likelihood		Possible			8–16
		Probable			>17

According to the understanding of the clinical symptoms and the pathophysiology of PSH, the therapeutic options rely on three main goals, namely, avoid triggers, reduce the clinical symptoms of the sympathetic outflow, and address the effects of PSH on other organs. Although this therapeutic strategy appears logical, there is no consensus in the literature given the small number of case series published. No drug is known to have a greater effect on PSH, and practically most patients require the use of several drugs based on local team approaches rather than objective scientific evidence [1,3-5].

Opioids are the first drug used, especially morphine or fentanyl, for their effect on nociceptive stimuli as well as on central pathways that involve paroxysms [1,3]. Temperature management can be achieved by methods such as surface cooling devices, antipyretic treatment, or endovascular technics [1]. Non-selective beta-blockers, especially propranolol due to its ability to pass the blood-brain barrier, might be used to decrease the severity and frequency of fever, diaphoresis, and dystonic postures by reducing the impact of circulating catecholamines [1]. Some studies have illustrated the use of bromocriptine (dopamine D2 agonists with a dose of 1.25 mg every 12 hours) for fever reduction and excessive sweating as they noted similarities between PSH patients and neuroleptic malignant syndrome caused by dopamine blockage [1,6]. Reports have shown the efficiency of daily use of gabapentin on PSH due to its effect on presynaptic voltage-gated calcium channels in the spinal cord and, therefore, inhibiting neurotransmitter release in the central nervous system [1]. The recommended dose is 3,600-4,800 mg/day [6]. No mention of the duration of treatment was found in the literature; it seems that it might depend on the approaches of each medical team. Finally, the authors agree that all these protocols might shorten the ICU length of stay and reduces the incidence of complications [1,3,6].

The outcomes and prognosis of PSH in TBI patients remain controversial. Although their association is frequent, the assertion that PSH is a prognosis factor for increased morbidity and poor clinical outcome is uncertain [3,9,10]. The literature agrees that early diagnosis may help improve morbidity in these patients by shortening the ICU length of stay and reducing long-term complications, such as pulmonary infections after a long duration of mechanical ventilation, tracheostomy, and, in worst cases, death [1,4,6]. Other studies have reported a low impact on morbidity and mortality which can be explained by the variety of symptoms and the sample size studied [4,9].

## Conclusions

PSH is a frequent but unknown clinical syndrome that appears after TBI. The definition and clinical criteria for its diagnosis were the first steps in a long journey to study the syndrome. The pathophysiology of PSH remains controversial, with the main theory accepted being the excitatory/inhibitory ratio model, which led to large potential therapies for this condition. Through this case report and literature review, we highlight the role of the pathophysiology in PSH syndrome, the clinical finding, and the diagnostic criteria, as well as discuss the different types of therapeutic options that physicians might have, even though no consensus was made. The main challenge in the PSH syndrome is to develop clear guidelines (through larger studies) concerning the treatment and define patient outcomes.
